# Predicting the geographical distribution of drug use disorder in Sweden from the geographical variation in social deprivation, genetic risk and urbanization

**DOI:** 10.1038/s41380-026-03636-x

**Published:** 2026-05-11

**Authors:** Kenneth S. Kendler, Pengxiang Zhao, Ali Mansourian, Henrik Ohlsson, Jan Sundquist, Bo Malmberg, Kristina Sundquist

**Affiliations:** 1 Virginia Institute for Psychiatric and Behavioral Genetics, Virginia Commonwealth University, Richmond, VA USA; 2 Department of Psychiatry, Virginia Commonwealth University, Richmond, VA USA; 3 Department of Physical Geography and Ecosystem Science, Lund University, Lund, Sweden; 4 Center for Primary Health Care Research, Lund University, Malmö, Sweden; 5 Department of Geography of Stockholm University, Stockholm, Sweden

**Keywords:** Genetics, Psychology

## Abstract

Drug use disorder (DUD) is distributed unevenly over geographical areas of Sweden. We explore, using geographically weighted regression, the proportion of this variation associated with community levels of genetic risk, social deprivation and urbanicity. In the 5983 Demographic Statistical areas (DeSOs) within Sweden, we predict DeSO prevalence from mean genetic risk for DUD, measured by the family genetic risk score (FGRS_DUD_), social deprivation (SD) and degree of urbanization. We first modeled results for genetic risk and SD only finding, in all of Sweden, the proportions of predicted variance (±95% CIs) in DeSO levels of DUD associated with genetic risk and SD were, respectively, 0.58 (0.58–0.59) and 0.42 (0.41–0.42). Similar results were obtained in the major cities: Stockholm, Gothenburg, and Malmö. Genetic effects were more predictive in males while levels of SD were more predictive in females. In maps of FGRS_DUD_ and SD prediction of DUD levels in DeSOs, we found results consistent with prior geographical patterns known in Sweden. Adding urbanicity to our model, only modestly predicted DeSO levels of DUD. To explore the potential origins of geographic variation in FGRS_DUD_, we showed that young adults with high FGRS_DUD_ preferentially migrated to areas in Sweden with high levels of DUD. Our findings provide an initial tentative step toward clarifying the role of genetic and social-demographic factors in the geographical distribution of DUD within Sweden.

Drug use disorder (DUD) is a multifactorial syndrome with important causal factors from biological/genetic, psychological, and social domains [1–3]. Genetic factors play an important role in the etiology of DUD [4] with heritability estimates around 50% [5, 6].

The geographical distribution of DUD in human populations is typically uneven, with areas of low and high prevalence [7–12]. The risk for illicit substance use and DUD is often associated with low levels of education, income, and employment, as well as residence in areas of socially deprivation, and often, but not always, urban environments [1, 7, 13–17]. With increased availability of genetic data, genetic risk factors for disorders can be mapped, showing spatial heterogeneity [18–21]. Indeed, we recently showed substantial geographical variation in genetic risk for DUD in Sweden [8].

In this paper, we seek to gain insight into the important question of the causes of the spatial variation of DUD in Sweden. We do this by examining the degree to which DUD prevalence is associated with the geographical variation of three potential risk factors for DUD: genetic liability, social deprivation (SD), and urbanization. Genetic liability is assessed by the family genetic risk score (FGRS), a quantitative measure of genetic risk based which has performed well in a number of empirical applications [22–28]. Our SD measure has been well validated and shown to be predictive of DUD in Sweden [29]. Our measure of urbanicity was developed by Statistics Sweden.

To reach this aim, we address four questions:

First, using geographically weighted regression (GWR) across the 5983 Demographic Statistical (DeSO) Areas within Sweden, we ask what is the mean proportion of explained variance in DUD prevalence in the entire country and in each of the three major metropolitan areas – Stockholm, Gothenburg, and Malmö –predicted by variation in FGRS_DUD_ versus by variation in levels of SD? We then ask whether these results differ meaningfully in men versus women.

Second, when we map these GWR findings, what geographical pattern appears for Sweden as a whole and its three major metropolitan areas? In particular, where in Sweden are rates of DUD more strongly predicted by variation in genetic risk versus by levels of social deprivation? Do these patterns relate to what is known about the human geography of the Swedish population.

Third, when we add an urbanicity measure to our regression, what further proportion of explained variance in DUD prevalence does it uniquely predict?

Fourth, can we obtain insights into the possible origin of the geographical variation in genetic risk factors for DUD within Sweden. In particular, can we show, using epidemiological statistical tools, whether internal migration patterns might explain some of this variation?

## Methods

We collected information on individuals from Swedish population-based registers with national coverage linking each person’s unique personal identification number which, to preserve confidentiality, was replaced with a serial number by Statistics Sweden. Ethical approval came from the Lund Regional Ethical Review Board, and no participant consent was required (No. 2008/409 and later amendments). For our definition of DUD, see Appendix Table S1. Our main analyses included individuals born in Sweden (1980–2000) (ages 15–35 in 2015) to Swedish-born parents, and for each individual, we included an FGRS for DUD. The FGRS is calculated from morbidity risks for disorders in first-degree through fifth-degree relatives, controlling for cohabitation effects, and thus arise from phenotypes in extended pedigrees, not from molecular genetic data (details in Appendix Table S2). Furthermore, we included registrations for DUD during a three-year period from 2016 onwards. For the first two aims, we included, for the year 2015, the DeSO area where the individual resided. DeSO areas divide Sweden into 5983 areas with between 700 and 2,700 inhabitants. The division considers the geographical conditions, so the boundaries typically follow streets, waterways, and railways. For analyses, we calculated the mean values of the FGRS_DUD_ and rates of DUD per DeSO. Additionally, for each DeSO, we created a neighborhood social deprivation (SD) index based on register data for residents aged 25–64. Four variables went into this index: low educational status (defined as less than 10  years of formal education); low income (from all sources was defined as less than 50% of individual median income); unemployment (defined as not employed; excluding full-time students, those in the military and retirees); and social welfare assistance. For urbanicity, we used a three-level index created by Statistics Sweden: a) rural areas; b) suburbs, and c) urban areas, typically divided into multiple DeSOs.

For the first aim, we used geographically-weighted regression (GWR) to model the association between DeSO rates of DUD and DeSO levels of FGRS_DUD,_ SD, and urbanicity. GWR extends traditional regression models to account for spatial heterogeneity and provides insights into how the strength and nature of relationships between dependent and independent variables change depending on geographic location. Compared with ordinary least squares regression, GWR recognizes the spatial variations in relationships across the space by estimating local, rather than global, regression coefficients [30]. To achieve this, a separate regression model at each location is fitted using a subset of nearby observations. The proximity of observations is typically weighted by a kernel function (e.g., Gaussian function), which assigns greater weight to observations closer to the location of interest and less weight to those farther away. A bandwidth parameter controls the extent of spatial influence and determines how many nearby observations contribute to the local regression. GWR has been widely used in various fields, including, but not limited to, geography and epidemiology [30–32].

To achieve our second aim, variance decomposition was further investigated, to examine how the geospatial patterns of DUD rates of DeSOs could be predicted by DeSO levels of FGRS_DUD_, SD and urbanicity. We estimated the percentage of variance in rates of DUD that was uniquely explained by each independent variable, as well as the proportion of variance attributed to the shared influence of multiple predictors [33]. As we are subdividing the explained variance from the GWR models, the sum across all the predictors will equal 100%. Since one linear regression model is developed for each DeSO area in GWR, we calculated the percentages of variance attributed to DeSO level FGRS, SD, and, in some models, urbanicity for each DeSO.

For the third aim, we used the population born 1975 to 1985 to predict rates of DUD and mean level of FGRS_DUD_ in the DeSO area at age 33 by individual level of FGRS_DUD_. We used a linear regression model with rates of DUD at the DeSO level (and mean level of FGRS FGRS_DUD_) as the outcome and FGRS_DUD_ at the individual level as the main predictor. We divided the individual FGRS into four groups, identified via K-means clustering. In Model A, we controlled for year of birth and rates of DUD (level of FGRS_DUD_) in the DeSO area at age 18. In Model B, we controlled for SD in the DeSO area at age 18 as well as an individual FGRS for education as a measure of individual socioeconomic status (for details, see Appendix Table S1). Only individuals that moved DeSO area between ages 18 and 33 were included (90.6% of the 435,410 males and 93.1% of the 401,665 females).

## Results

### Sources of variation of rates of drug use disorder across DeSOs with Sweden

We begin with Model 1, including the predictors FGRS_DUD_ and SD, which explained 25.7% (± 95% CIs; 23.2–28.5) of the total variance in DUD across DeSOs within Sweden. As depicted in Table 1, when we decompose that explained variance for all of Sweden, the mean level of FGRS_DUD_ and SD (±95% CIs) explain, respectively, 58% (58–59) and 42% (41–42) of that variance. We then fit the same model to Sweden’s three major metropolitan areas: Stockholm, Gothenburg, and Malmö finding results similar to those obtained in the national sample.

**Table 1:** Mean and 95% confidence intervals of the variance of Drug Use Disorder by DeSO Explained by Variance in Genetic Risk, Social Deprivation and Urbanization in the Country of Sweden and its Three Major Metropolitan Areas.

Area	Mean Proportion of Variance (± 95% CIs) in DeSO’s								
	Entire Sample			Males			Females		
	FGRS_DUD_	Social Deprivation	Urbanization	FGRS_DUD_	Social Deprivation	Urbanization	FGRS_DUD_	Social Deprivation	Urbanization
Sweden Model 1	0.58 (0.58–0.59)	0.42 (0.41–0.42)	--	0.66 (0.66–0.67)	0.34 (0.33–0.34)	--	0.51 (0.50–0.51)	0.49 (0.49–0.50)	--
Sweden Model 2	0.52 (0.46–0.59)	0.37 (0.31–0.43)	0.11 (0.08–0.13)	0.57 (0.48–0.65)	0.32 (0.24–0.41)	0.11(0.08–0.14)	0.45 (0.36–0.54)	0.45 (0.36–0.55)	0.10 (0.07–0.14)
Stockholm Model 1	0.57 (0.56–0.58)	0.43 (0.42–0.44)	–	0.65 (0.64–0.66)	0.35 (0.35–0.36)	–	0.48 (0.47–0.50)	0.52 (0.50–0.53)	–
Gothenburg Model 1	0.60 (0.59–0.62)	0.40 (0.38–0.41)	–	0.58 (0.57–0.59)	0.42 (0.41–0.43)	–	0.48 (0.49–0.50)	0.52 (0.51–0.53)	–
Malmö Model 1	0.57 (0.57–0.57)	0.43 (0.43–0.43)	–	0.74 (0.73–0.74)	0.26 (0.26–0.27)	–	0.45 (0.44–0.45)	0.55 (0.50–0.56)	–
Model 1 - Genetic Risk and Social Deprivation; Model 2 Genetic Risk,Social Deprivation and Urbanization.									

However, when analyzed separately by sex, we saw consistent differences between men and women. Across all of Sweden and its three major metropolitan areas, FGRS_DUD_ accounted for an appreciably higher proportion of the spatial variation in DUD in males than in females. For example, in the entire country, genetic risk accounted for about two-thirds of the variation in DUD levels in men, but almost half in females. The pattern of sex differences was consistent across the three metropolitan areas, but differed in magnitude most in Malmö and least in Gothenburg.

We then fitted Model 2 to the national data – containing FGRS_DUD_, SD, and urbanicity – and this model explained 27.3% (24.9–30.0) of the total variance in DUD across DeSOs, a modest increase from Model 1. For all of Sweden, we saw the percent of explained variance from FGRS_DUD_, SD, and urbanicity equaled 52% (46–59), 37% (31–43), and 11% (8–13), respectively. As seen in Model 1, sex differences persisted in Model 2 with greater variance in males explained by genetic risk and the opposite in females.

### Mapping areas of Sweden by the proportion of variance in DUD that arises from genetic factors versus social deprivation

**Fig. 1:**
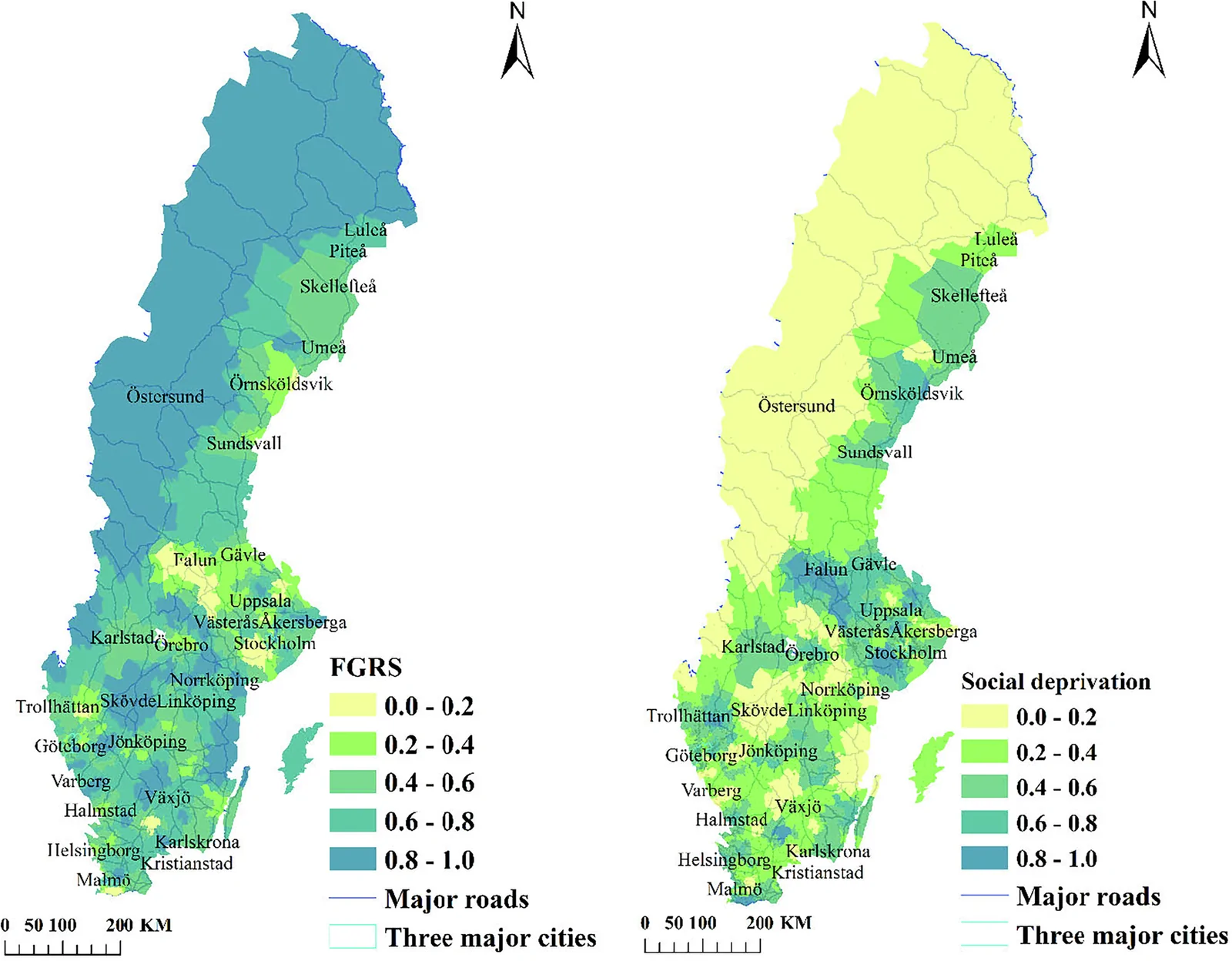
Spatial distribution of the proportion of variation in DUD in 2015 per DeSO in Sweden explained by variance in the Family Genetic Risk Score (FGRS) for DUD (left figure) and variance in social deprivation (right figure). The proportions of variance in between DeSO differences accounted for by the Family Genetic Risk Score for DUD (left figure) and by social deprivation (right figure) per individual DeSO are depicted by five color-coded categories from yellow for 0–20% up to blue for 80–100% of the variance.

**Fig. 2:**
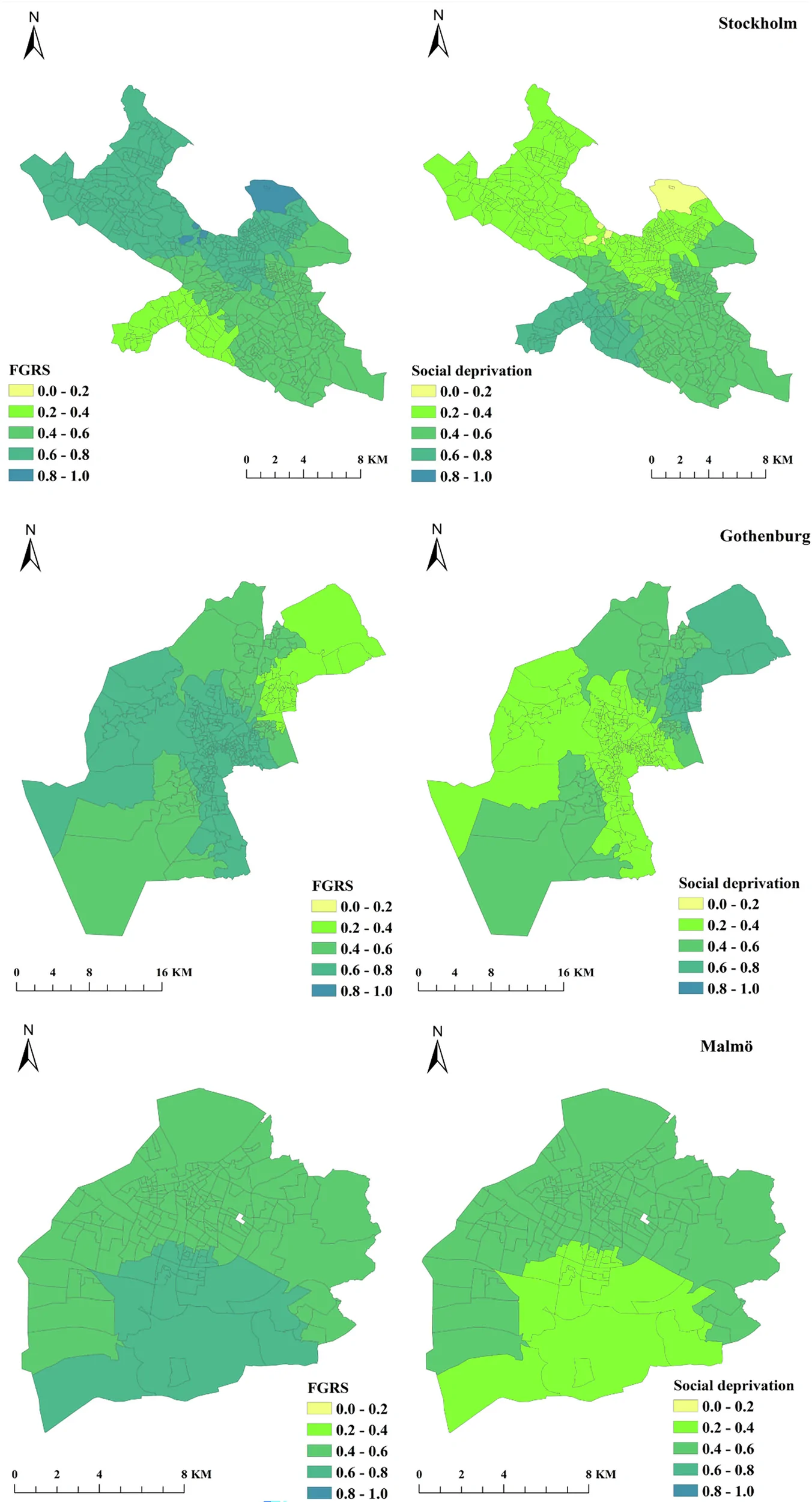
Spatial distribution of the proportion of variation in DUD in 2015 per DeSO in the three major Swedish cities -- Stockholm, Gothenburg, and Malmö – from top to bottom explained by variance in the Family Genetic Risk Score (FGRS) for DUD (left figure) and variance in social deprivation (right figure). The proportions of variance in between DeSO differences accounted for by the Family Genetic Risk Score for DUD (left figure) and by social deprivation (right figure) per individual DeSO are depicted by five color-coded categories from yellow for 0–20% up to blue for 80–100% of the variance.

Given the similarity of results in our two models and the greater ease of mapping two versus three predictors, we present, in Figs. 1 and 2, heat maps for, respectively, all of Sweden and Sweden’s three largest metropolitan regions as a function of the proportion of variation in DeSO-level rates of DUD that are associated with spatial variation in genetic risk and SD scores. Since there are only two predictors in the model, by definition, the sum of the two proportions equals unity. Thus, in DeSOs with the lightest shade of blue, variation in FGRS_DUD_ accounts for only 0–20% of the variation in DUD levels, while variation in SD accounts for 80–100%. Nationally, in Fig. 1, we see rural DeSOs in Western and Northern Sweden where the large proportion of DeSO level differences in rates of DUD was driven by differences in genetic risk. Results in Northeastern Sweden along the Baltic were more variable, as were results in the southern third of Sweden. Noteworthy was one relatively large area northwest of Stockholm, where large proportions of the differences in DUD rates in DeSOs were driven by variation in levels of SD.

**Fig. 3:**
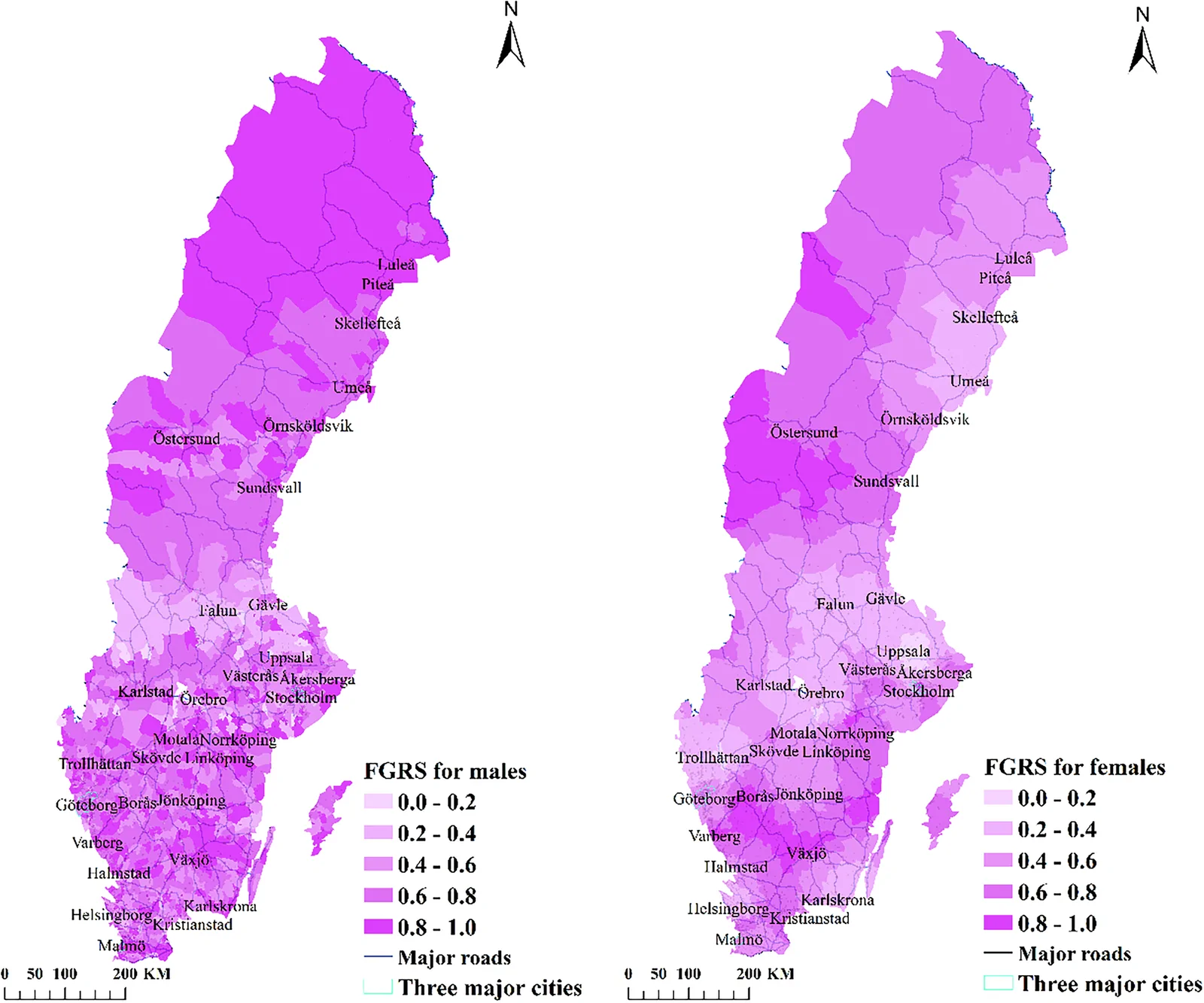
Spatial distribution of the proportion of variation in DUD in 2015 per DeSO in Sweden explained by variance in the Family Genetic Risk Score (FGRS) for DUD in Males (left figure) and Females (right figure). The proportions of variance in between DeSO differences accounted for by the Family Genetic Risk Score for DUD are depicted by five color-coded categories from yellow for 0–20% up to blue for 80–100% of the variance.

Patterns were more distinctive for the three major metropolitan areas. For Stockholm (Fig. 2 - top), in areas to the North and Northwest, differences in DeSO levels were largely explained by differences in genetic risks. By contrast, for districts in the east and south of the city, variations in DUD levels were more closely related to differences in SD. In Gothenburg (Fig. 2 - middle), DeSO variation in DUD levels were largely associated with genetic effects in the central region of the metropolitan area but by levels of SD in the southern and north-eastern areas. Malmö (Fig. 2 – bottom) had less extreme variations in the roles SD and FGRS_DUD_. We present in Fig. 3, the spatial distribution in Sweden of the proportion of variation in DUD explained by variance in FGRS_DUD_ in males (left figure) and females (right figure).

Finally, we examined internal migration patterns within Sweden, predicting from an individual’s level of FGRS_DUD_ the frequency of DUD in the DeSO in which they resided at the age of 33, controlling for year of birth and the frequency of DUD in the DeSO in which they resided at age 18 (Table 2). In both males and females, we divided their mean level of FGRS_DUD_ into quartiles, using the lowest quartile as a reference in the regression model. Males in the highest quartile of genetic risk for DUD moved into areas where the percentage of population that had DUD was 1.83% (95% CIs 1.72–1.94) higher. We then added controls for the level of social deprivation in the DeSO they lived in at 18 and their genetic risk for low educational attainment. This reduced this effect size to 1.65% (1.54–1.76) higher rates of DUD. The pattern of these results was similar in women, but the magnitudes of the effects were smaller.

**Table 2:** Internal Migration Patterns within Sweden as a Function of the Genetic Risk for Drug Use Disorders*.

MALES	Rates of DUD		Level of FGRS_DUD_	
FGRS_DUD_	Beta (95% CI)	Beta (95% CI)	Beta (95% CI)	Beta (95% CI)
High	1.83 (1.72; 1.94)	1.65 (1.54; 1.76)	0.06 (0.06; 0.07)	0.06 (0.05; 0.06))
Mid High	0.82 (0.78; 0.87)	0.71 (0.66; 0.75)	0.03 (0.03; 0.03)	0.03 (0.02; 0.03)
Mid Low	0.44 (0.41; 0.47)	0.35 (0.32; 0.38)	0.02 (0.02; 0.02)	0.01 (0.01; 0.02)
Low	Reference	Reference	Reference	Reference
YoB	0.05 (0.05; 0.06)	0.05 (0.05; 0.06)	0.00 (0.00; 0.00)^NS^	0.00 (0.00; 0.00) ^NS^
DUD/FGRS at age 18	0.18 (0.17; 0.18)	0.18 (0.17; 0.18)	0.25 (0.25; 0.26)	0.26 (0.26; 0.27)
FGRS_Education_		0.24 (0.23; 0.26)		0.01 (0.01; 0.01)
Deprivation at age 18		−0.01 (−0.03; −0.00)*		−0.01 (−0.01; −0.01)

MALES	Rates of DUD		Level of FGRS_DUD_	
FGRS_DUD_	Beta (95% CI)	Beta (95% CI)	Beta (95% CI)	Beta (95% CI)
High	1.20 (1.11; 1.28)	1.06 (0.98; 1.15)	0.05 (0.05; 0.06)	0.05 (0.04; 0.05)
Mid High	0.68 (0.65; 0.72)	0.59 (0.55; 0.63)	0.03 (0.03; 0.03)	0.03 (0.02; 0.03)
Mid Low	0.35 (0.32; 0.37)	0.27 (0.24; 0.29)	0.02 (0.01; 0.02)	0.01 (0.01; 0.01)
Low	Reference	Reference		
YoB	0.05 (0.05; 0.05)	0.05 (0.05; 0.05)	0.00 (0.00; 0.00) ^NS^	0.00 (0.00; 0.00) ^NS^
DUD/FGRS at age 18	0.16 (0.16; 0.17)	0.17 (0.16; 0.17)	0.24 (0.24; 0.24)	0.26 (0.25; 0.26)
FGRS_Education_		0.20 (0.19; 0.21))		0.01 (0.01; 0.01)
Deprivation at age 18		−0.04 (−0.05; −0.03)		−0.01 (−0.01; −0.01)

All *P*-values were <0.0001 expect: * 0.01–0.05; and NS:P > 0.05.

*Individuals born in Sweden (1975–1985) to Swedish born parents. Predicting DUD rates and levels of FGRS in DESO area at ages 33. Only individuals that moved are included.

We then repeated those results using an identical model, but now predicting level of FGRS_DUD_ in the DeSO where they resided at age 33. The effect size is in SD units. In males, controlling for all the covariates, those in the highest quartile of level of FGRS_DUD_ from ages 18–33 were living in a DeSO with a mean level of FGRS_DUD_ which was +0.06 (0.06–0.07) SD higher. For females, the effect size was only slightly smaller (0.05 SDs).

## Discussion

This report sought to clarify the causes of the geographical distribution of DUD in Sweden [8] using geographically weighted regression with three predictors each assessed at the DeSO level: FGRS_DUD_, SD, and urbanicity. Prior studies of geographical variation in illicit substance use or DUD have largely focused on social determinants [1, 7, 13–15], although the possible role of genetic risk factors has been noted [11]. We are unaware of prior research seeking to explain geographic variation of DUD jointly from genetic and social variables. Our analyses build on prior findings in Sweden that rates of both DUD [8] and FGRS_DUD_ are quite spatially heterogeneous [34]. We focus here on six of the specific findings in this report that we regard of broad importance. We comment on each of them.

First, we found that the variation in rates of DUD in DeSOs across all of Sweden was more strongly predicted by mean DeSO level of genetic risk than by DeSO level of SD. Given the magnitude of genetic influences on risk with DUD, with heritability estimates typically of around 50% [5, 6], and the substantial spatial variation recently shown in Sweden for this genetic risk [34], these results are not surprising. However, if they generalize beyond Sweden, they do suggest that some broadening of perspective in drug abuse epidemiologic studies of the spatial distribution of DUD, which have typically focused strongly on the social determinants of DUD, would be helpful.

Second, the findings across all of Sweden – that around three-fifths of the variation in DeSO level of DUD could be explained by variation in FGRS_DUD_, and around two-fifths by levels of SD – were quite similar to that seen in Sweden’s three major metropolitan areas: Stockholm, Gothenberg, and Malmö. This occurred despite the fact that each of these urban areas contained DeSOs with quite variable levels of genetic and social risks for DUD.

Third, we saw consistent differences across sexes in the results of our GWR in all our analyses. Nationwide, while genetic risk accounted for almost exactly two-thirds of the DeSO variation in rates of DUD in men, in women, the results were approximately one-half. Two large scale twin sibling studies of DUD in Sweden have been performed, one of which, consistent with our finding here, showed higher levels of heritability for DUD in men than in women [35], while the other study showed the reverse [29]. While there is a large literature on sex differences in DUD [36], we are not aware of prior studies suggesting sex differences on the impact of SD on DUD. There is, however, some evidence for greater sensitivity to the impact of psychosocial adversity in women versus men. In a large national U.S. epidemiological survey, risk for cannabis use disorder much more strongly predicted both childhood and current adversities in women than in men [37]. Further work is needed to understand sex-based differences in the impact of genetic and social risk factors in Sweden.

Fourth, given widespread interest in the impact of urbanicity on human health [38, 39], we inquired whether measure of urbanicity would add substantial predictive power to our basic model. Its predictive power was modest in our hands, increasing the explained variance from 25.7 to 27.3%. It changed little the relative magnitude of the predictive power of FGRS_DUD_ versus SD.

Fifth, we wanted, in this manuscript, to explore possible mechanisms responsible for the geographical variations of the mean DeSO FGRS_DUD_ in Sweden. Our model – focused on differential migration patterns within the country based on an individual’s FGRS_DUD_ – was meant to be more illustrative than definitive. But it clearly shows that a high genetic risk for DUD, in both males and females, predicted internal migration within Sweden. Individuals with a high level of FGRS_DUD_, between the ages of 17 and 33, were prone to move to DeSOs with high rates of DUD and, albeit less strongly, high mean rates of FGRS_DUD_. The degree to which this mechanism can explain the current distribution of genetic risk for DUD in Sweden is a question for further analyses. Our results are broadly comparable to those of Abdellaoui et al., who found in Great Britain that the pattern of clustering of the genetic potential for educational attainment was probably the result of migration driven by socioeconomic status [20].

Finally, while our approach was by design descriptive, it is worth inquiring what potential causal effects could underlie the pattern of our findings. At an individual level, pathways to DUD are likely etiologically heterogeneous. For example, in a prior study based on detailed information from the conscript registry, a structural model containing 20 risk factors and explaining almost 50% of the total variance in DUD, we uncovered two major connecting pathways that reflected i) genetic/familial risk and ii) family dysfunction and psychosocial adversity [1]. It is plausible that these pathways might not only differentiate between individuals but between communities. We might speculate that in more urbanized areas in Sweden, where variation in drug supply and stress-induced drug use are high, SD could play a particularly strong role in the variation in the community level of DUD. We see evidence consistent with this hypothesis in South-West Stockholm and in North-East Gothenburg. By contrast, in the large rural areas of central and north-western Sweden, where social conditions are more homogeneous and drug availability likely lower, genetically driven personality differences might play a more prominent role in the community rates of DUD. We also see this pattern in less urbanized peripheral areas with smaller local variation in deprivation, in homogenous middle-class areas on the outskirts of the largest cities, and in the central parts of Gothenburg.

One of us (BM) has developed a multiscale cluster typology of ten types of residential areas in Sweden [40] (five types each in a rural versus urban setting). One important way in which these areas are characterized is by their level of diversity versus homogeneity. We would speculate that homogeneous rural and urban areas would tend to be places where genetic effects play a strong role in predicting variation in DUD. By contrast, diverse rural and urban areas would be locations where SD was the larger driving force in differences in DeSO levels of DUD. While beyond the scope of the present set of analyses, a rough correspondence can be seen between this cluster typology and the patterns we see in Figs. 1 and 2.

Our work has potential implication for efforts at DUD prevention. One of the many methods that have been tried has been relocation of families from areas of high to low SD. A classical example of this was the Moving to Opportunity program where households with children living in high poverty public housing projects in five cities in the US were offered housing vouchers by lottery to support living in neighborhoods with lower levels of SD [41]. The outcomes for illicit substance use in this study were mixed [42]. This prevention approach was predicated on the assumption that by moving from communities of high to low SD, rates of DUD would decline because the high rates of DUD in communities with high levels of SD were largely social in origin. Our findings -- that a substantial proportion of the high concentration of DUD in certain communities -- was a result of differences in population genetic risk and not levels of SD, might lead to less optimistic predictions about the preventative effects of relocation.

## Limitations

These results should be interpreted in the context of four potential major methodological limitations of this work. First, the value of this report depends on the validity of our assessment of DUD by registry data which is supported by high cross-method agreement [43] and by close resemblance of our genetic epidemiological findings for substance use disorders in Sweden to those obtained in other samples [29, 43–45].

Second, the FGRS is an index of risk based on the phenotypes of members of the extended family and is not comparable to polygenic risks scores obtained from molecular genetic samples. The FGRS has performed well in empirical applications with simulations and comparisons with other similar methods [46], suggesting that it provides a good measure of genetic risk [22–28]. Furthermore, recent empirical comparisons for polygenic risk scores and related simulation studies, suggest that both FGRS-like phenotypic measures and polygenic risk scores, with available samples, are best seen as modestly accurate measures of the same underlying genetic susceptibility [47, 48].

Third, the predictive power of our geographically weighted regression model for DUD varied across Sweden. We present these results in Appendix Figures S1–2. Because we were interested in the proportion of this variance predicted by FGRS_DUD_ versus SD, we did not focus on this variation in the present report.

Finally, our analyses – associating FGRS_DUD_, SD and urbanicity measures with DeSO prevalences of DUD – are essentially correlations. No formal causal inference methods were employed. At the individual level, the causal pathways connecting these measures to DUD are long and complex, with multiple mediating variables [1]. When moving from the individual to the community level, we expect the pathways to be even more complex and likely interwoven. This report which predicts the prevalence of the complex human syndrome for DUD in small geographical areas in Sweden by the additive effects of just three high level variables (FGRS_DUD_, SD and urbanicity) is just a first tentative step in a long empirical project to understand the interrelationship between individual and community level risk factors for DUD in Sweden.

## Conclusions

We sought to clarify the degree to which variation in the DUD prevalence in Swedish communities could be predicted by variance in the mean FGRS_DUD_, SD, and urbanization in those DeSOs. Across all of Sweden and in its three major metropolitan areas, in our full model, roughly 50% of that explained variance was due to FGRS_DUD_, 40% to SAD, and 10% to urbanization. Genetic effects were consistently more predictive in males than females, while the reverse pattern was seen for levels of SD. We provided preliminary evidence for a mechanism to account for the variation in FGRS_DUD_ across Sweden wherein young adults with high levels of FGRS_DUD_ preferentially migrated to areas with higher levels of DUD and FGRS_DUD._ This report represents a first step toward clarifying the role of genetic and social-demographic factors in the geographical clustering of DUD within Sweden.

## Supplementary information

Appendix

## Data Availability

The data for this study are not publicly available due to legal restrictions with regard to the nationwide Swedish registers, but they can be acquired directly from the responsible authorities pending their approval. For an instruction document about our model implementation and the Python code used, see https://doi.org/10.5281/zenodo.17641294.

## Data Analysis

Kristina Sundquist MD PhD had full access to all the data in the study and takes responsibility for the integrity of the data and the accuracy of the data analysis.
